# *Bacillus licheniformis* Contains Two More PerR-Like Proteins in Addition to PerR, Fur, and Zur Orthologues

**DOI:** 10.1371/journal.pone.0155539

**Published:** 2016-05-13

**Authors:** Jung-Hoon Kim, Chang-Jun Ji, Shin-Yeong Ju, Yoon-Mo Yang, Su-Hyun Ryu, Yumi Kwon, Young-Bin Won, Yeh-Eun Lee, Hwan Youn, Jin-Won Lee

**Affiliations:** 1 Department of Life Science and Research Institute for Natural Sciences, Hanyang University, Seoul, 04763, Republic of Korea; 2 Department of Biology, California State University Fresno, Fresno, California, 93740–8034, United States of America; Loyola University Chicago, UNITED STATES

## Abstract

The ferric uptake regulator (Fur) family proteins include sensors of Fe (Fur), Zn (Zur), and peroxide (PerR). Among Fur family proteins, Fur and Zur are ubiquitous in most prokaryotic organisms, whereas PerR exists mainly in Gram positive bacteria as a functional homologue of OxyR. Gram positive bacteria such as *Bacillus subtilis*, *Listeria monocytogenes* and *Staphylococcus aureus* encode three Fur family proteins: Fur, Zur, and PerR. In this study, we identified five Fur family proteins from *B*. *licheniformis*: two novel PerR-like proteins (BL00690 and BL00950) in addition to Fur (BL05249), Zur (BL03703), and PerR (BL00075) homologues. Our data indicate that all of the five *B*. *licheniformis* Fur homologues contain a structural Zn^2+^ site composed of four cysteine residues like many other Fur family proteins. Furthermore, we provide evidence that the PerR-like proteins (BL00690 and BL00950) as well as PerR_BL_ (BL00075), but not Fur_BL_ (BL05249) and Zur_BL_ (BL03703), can sense H_2_O_2_ by histidine oxidation with different sensitivity. We also show that PerR2 (BL00690) has a PerR-like repressor activity for PerR-regulated genes *in vivo*. Taken together, our results suggest that *B*. *licheniformis* contains three PerR subfamily proteins which can sense H_2_O_2_ by histidine oxidation not by cysteine oxidation, in addition to Fur and Zur.

## Introduction

The ferric uptake regulator (Fur) protein is an iron-sensing transcriptional regulator which controls the expression of genes involved in intracellular iron homeostasis [1]. Under iron-replete conditions, Fur mediates the repression of genes involved in intracellular iron increase to prevent iron overload. Since its first recognition in *Escherichia coli*, Fur family proteins have been found and characterized in a variety of organisms ranging from bacteria to archaea [1, 2]. Fur family proteins are not only responsible for the acquisition and storage of iron, but also involved in the oxidative stress response as well as in the acquisition and storage of other metal ions. Now it is appreciated that there are various subgroups of Fur family proteins, which include zinc uptake regulator (Zur), manganese uptake regulator (Mur), nickel uptake regulator (Nur), heme-dependent iron response regulator (Irr) and metal-dependent peroxide regulon repressor (PerR), in addition to Fur itself [1, 2].

Fur family proteins are homo dimeric DNA binding proteins, and each monomer is composed of two domains, a winged-helix DNA-binding domain at the N-terminus and a dimerization domain at the C-terminus, connected by a loop. The available structures of Fur family proteins indicate that many, but not all, of these proteins have at least two metal binding sites in each monomer: a structural Zn^2+^-binding site and a regulatory metal-binding site [3–9]. The structural Zn^2+^ is usually coordinated by four Cys residues arranged in two CXXC motifs and stabilizes the dimerization domain. The regulatory metal binding site, located in the hinge region between the DNA binding domain and the dimerization domain, engages amino acids from both domains. In Fur and PerR proteins, the regulatory metal binding site is penta- or hexa-coordinated by three His residues and two Asp/Glu residues [4, 6, 8, 9], whereas this site is tetra-coordinated by two His residues, one Asp/Glu residue, and one Cys residue in Zur proteins [3, 5, 7].

While Fur and Zur are widely distributed in both Gram positive and Gram negative bacteria, PerR is mainly found in Gram positive bacteria as a functional homologue of OxyR [10]. PerR regulates genes involved in oxidative stress response like OxyR. However, unlike cysteine-thiol based peroxide sensor OxyR, PerR senses H_2_O_2_ by Fe-mediated histidine oxidation [11]. Reaction of H_2_O_2_ with Fe^2+^ leads to a rapid oxidation of either one of the two His residues involved in Fe^2+^-coordination (His37 and His91 in *B*. *subtilis* PerR), resulting in the loss of repressor activity. However, Fur does not react with H_2_O_2_, despite the strong similarity of regulatory metal binding site [11, 12].

*B*. *licheniformis* is a Gram-positive, spore-forming soil bacterium which is closely related to the well-studied model organism *B*. *subtilis* [13]. *B*. *licheniformis* is an industrially important organism widely used for the manufacture of enzymes, peptide antibiotics and specialty chemicals. Despite the importance of stress physiology during the fermentation process, little is known about the physiology and stress response of *B*. *licheniformis* compared with its close relative *B*. *subtilis* [14].

Here we investigated the Fur family proteins from *B*. *licheniformis*. *B*. *licheniformis* genome encodes five Fur family proteins, with three of them clustering in the PerR group. We have unequivocally identified BL00075, BL03703, and BL05249 as PerR, Zur, and Fur, respectively, using *B*. *subtilis* promoter fusion reporter systems. In addition, we provide several lines of evidence that two novel PerR-like proteins, BL00690 and BL00950, are also able to sense H_2_O_2_ by histidine oxidation, and that BL00690 has a transcriptional repressor activity for PerR-regulated genes.

## Materials and Methods

### Bacterial strains and culture conditions

The bacterial strains used in this study are described in Table 1. *E*. *coli* and *B*. *subtilis* strains were routinely grown in Luria-Bertani (LB) media at 37°C with appropriate antibiotics. MOPS buffered minimal medium was used for the metal-limited minimal media (MLMM) as described previously [15]. Ampicillin (100 μg/ml) and chlorampenicol (34 μg/ml) were used for the selection of *E*. *coli* strains. Spectinomycin (100 μg/ml), erythromycin (1 or 5 μg/ml), neomycin (25 μg/ml), kanamycin (20 μg/ml), chlorampenicol (10 μg/ml), tetracyclin (10 μg/ml), and lincomycin (8 μg/ml) were used for the selection of *B*. *subtilis* strains. For the induction of *xylA* promoter, 1% xylose (w/v) was used.

**Table 1:** Bacterial strains used in this study.

Strains	Relevant genotype or purpose	Reference or source
*B*. *subtilis*		
HB9700	CU1065 *zur*::*tet*	[26]
HB9703	CU1065 *perR*::*tet*	[15]
HBL100	CU1065 *fur*::*kan*	This study
LB1066	CU1065 *fur*::*kan*, *zur*::*tet*, *perR*::*cat*	This study
LB1532	HB9703 *amyE*::*spc*, *SPβ2Δ2*::*Tn917*::*Φ(mrgA-cat-lacZ)*	[17]
HB9738	HB9703 *amyE*::*perRBS-FLAG*, *SPβ2Δ2*::*Tn917*::*Φ(mrgA-cat-lacZ)*	[15]
LB1023	HB9703 *amyE*::*bl00075-FLAG*, *SPβ2Δ2*::*Tn917*::*Φ(mrgA-cat-lacZ)*	This study
LB1034	HB9700 *amyE*::*spc*, *SPβ2Δ2*::*Tn917*::*Φ(yciC-cat-lacZ)*	This study
LB1035	HB9700 *amyE*::*zurBS-FLAG*, *SPβ2Δ2*::*Tn917*::*Φ(yciC-cat-lacZ)*	This study
LB1036	HB9700 *amyE*::*bl03703-FLAG*, *SPβ2Δ2*::*Tn917*::*Φ(yciC-cat-lacZ)*	This study
LB1040	HBL100 *amyE*::*spc*, *SPβ2Δ2*::*Tn917*::*Φ(feuA-cat-lacZ)*	This study
LB1041	HBL100 *amyE*::*furBS-FLAG*, *SPβ2Δ2*::*Tn917*::*Φ(feuA-cat-lacZ)*	This study
LB1042	HBL100 *amyE*::*bl05249-FLAG*, *SPβ2Δ2*::*Tn917*::*Φ(feuA-cat-lacZ)*	This study
LB1227	LB1066 *thrC*::*spc*	This study
LB1287	LB1066 *thrC*::*P*_*xylA*_*-bl00950-FLAG*	This study
LB1288	LB1066 *thrC*::*P*_*xylA*_*-bl00690-FLAG*	This study
LB1490	LB1066 *thrC*::*P*_*xylA*_*-perRBS-FLAG*	This study
LB1491	LB1066 *thrC*::*P*_*xylA*_*-furBS-FLAG*	This study
LB1493	LB1066 *thrC*::*P*_*xylA*_*-zurBS-FLAG*	This study
LB1233	LB1227 *SPβ2Δ2*::*Tn917*::*Φ(mrgA-cat-lacZ)*	This study
LB1234	LB1227 *SPβ2Δ2*::*Tn917*::*Φ(feuA-cat-lacZ)*	This study
LB1235	LB1227 *SPβ2Δ2*::*Tn917*::*Φ(yciC-cat-lacZ)*	This study
LB1297	LB1287 *SPβ2Δ2*::*Tn917*::*Φ(mrgA-cat-lacZ)*	This study
LB1288	LB1287 *SPβ2Δ2*::*Tn917*::*Φ(feuA-cat-lacZ)*	This study
LB1299	LB1287 *SPβ2Δ2*::*Tn917*::*Φ(yciC-cat-lacZ)*	This study
LB1300	LB1288 *SPβ2Δ2*::*Tn917*::*Φ(mrgA-cat-lacZ)*	This study
LB1301	LB1288 *SPβ2Δ2*::*Tn917*::*Φ(feuA-cat-lacZ)*	This study
LB1302	LB1288 *SPβ2Δ2*::*Tn917*::*Φ(yciC-cat-lacZ)*	This study
LB4031	LB1490 *SPβ2Δ2*::*Tn917*::*Φ(mrgA-cat-lacZ)*	This study
LB4065	LB1491 *SPβ2Δ2*::*Tn917*::*Φ(feuA-cat-lacZ)*	This study
LB4066	LB1493 *SPβ2Δ2*::*Tn917*::*Φ(yciC-cat-lacZ)*	This study
LB1010	HB9703 *amyE*::*spc*	This study
LB2128	HB9703 *amyE*::*P*_*perR*_*-NdeI-perRBS-FLAG*	This study
LB4034	HB9703 *amyE*::*P*_*perR*_*-NdeI-bl00690-FLAG*	This study
LB4106	HB9703 *amyE*::*P*_*perR*_*-NdeI-bl00950-FLAG*	This study
*E*. *coli*		
LE0001	BL21(DE3)pLysS pET-11a::*bl00950*	This study
LE0002	BL21(DE3)pLysS pET-16b::*bl00690*	This study
LE0008	BL21(DE3)pLysS pET-11a::*bl00075*	This study
LE0009	BL21(DE3)pLysS pET-11a::*bl05249*	This study
LE0010	BL21(DE3)pLysS pET-11a::*bl03703*	This study
LE1374	BL21(DE3)pLysS pET-15b::*His6-bl00950*	This study

### Construction of *E*. *coli* strains overexpressing Fur family proteins

The open reading frames (ORFs) of *bl05249*, *bl03703*, *bl00075*, *bl00950* and, *bl00690* were PCR-amplified with *B*. *licheniformis* ATCC14580 chromosomal DNA as template. The PCR fragments of *bl05249*, *bl03703*, *bl00075*, and *bl00950* were individually cloned into the *Nde*I and *Bam*HI sites of expression vector pET-11a (Novagen) resulting in plasmids named pJL303, pJL304, pJL302, and pJL201, respectively. The PCR fragments of *bl00690* were cloned into the *Nco*I and *Bam*HI sites of expression vector pET-16b (Novagen) resulting in plasmid named pJL202. For the purification of N-terminally His-tagged BL00950, the PCR-fragments of *bl00950* were cloned into *Nde*I and *Bam*HI sites of pET-15H-*oxyR* [16] resulting in plasmids named pJL853. The plasmids were introduced into *E*. *coli* BL21 (DE3) pLysS cells for the overexpression of encoded proteins.

Each *E*. *coli* BL21 (DE3) pLysS strain carrying pJL303, pJL304, pJL302, pJL853, or pJL202 was grown in 1 L of LB medium containing 0.4% (w/v) glucose, chloramphenicol, and ampicillin. At OD_600_ of ~0.4, isopropyl-β-D-thiogalactopyranoside (IPTG) was added to a final concentration of 1 mM (with additional final 50 μM ZnSO_4_ for cells expressing BL03703), and the cells were allowed to grow for an additional 2 h. The cells were harvested by centrifugation, and lysed by sonication for protein purification. BL00075, BL05249, and BL03703 were purified by heparin-Sepharose and MonoQ chromatography using buffer A (20 mM Tris-HCl, pH 8.0, 0.1 M NaCl, and 5% glycerol (v/v)) containing 10 mM EDTA for BL00075 and BL05249, or 2 mM EDTA for BL03703 with the application of a linear gradient of 0.1–1 M NaCl as described previously [15]. BL00690 was purified by heparin-Sepharose and SP-Sepharose chromatography using buffer A containing 10 mM EDTA with the application of a linear gradient of 0.1–1 M NaCl. Since BL00950 did not bind to heparin-Sepharose resin unlike other Fur family proteins, we used His-tagged BL00950 for this study. His-tagged BL00950 was first purified by Ni-NTA chromatography, and subsequently by SP-Sepharose chromatography using buffer A containing 10 mM EDTA with the application of a linear gradient of 0.1–1 M NaCl. All the proteins were further purified using a Superdex 200 HiLoad 16/60 column (GE Healthcare) equilibrated with Chelex-100-treated buffer A. Note that BL00950 was purified as monomer whereas all the other proteins were purified as dimers as judged by elution profiles from Superdex 200 HiLoad gel filtration chromatography. The purities of all of the purified proteins were checked by SDS-PAGE, and their concentrations were determined by measuring *A*_280 nm_ using the calculated values of molar extinction coefficient of each protein (BL05249: 11,460 M^-1^cm^-1^, BL03703: 10,430 M^-1^cm^-1^, BL00075: 8,940 M^-1^cm^-1^, BL00690:10,430 M^-1^cm^-1^, BL00950: 8,940 M^-1^cm^-1^).

### Electrophoretic mobility shift assay

The 431 bp DNA fragment containing *B*. *subtilis mrgA* promoter region was generated by PCR, and subsequently digested with *Eco*RI, resulting in a 273 bp fragment containing PerR box and a 154 bp fragment used for a non-specific control. The DNA fragments were end-labelled with [γ-^32^P] ATP using T4 polynucleotide kinase (NEB) and unincorporated labels were removed using nucleotide removal kit (Qiagen). Protein (BL00690 or BL00950) and a labelled probe were mixed in binding buffer (20 mM Tris-HCl pH 8.0, 50 mM KCl, and 5% glycerol (v/v), 50 μg/ml BSA and 100 μM MnCl_2_), and separated by 6% PAGE with a 45 mM Tris-borate buffer containing 100 μM MnCl_2_. After 2 h at 120 V, the gel was dried and exposed to X-ray film with an intensifying screen (Kodak) at -80°C.

### Measurement of Zn^2+^ release by H_2_O_2_ using PAR

Measurement of Zn^2+^ release by H_2_O_2_ was performed as described previously [15, 17]. 5 μM protein in buffer A was treated with 0, 1, 10, or 100 mM H_2_O_2_ in the presence of 100 μM 4-(2-pyridylazo)resorcinol (PAR), and Zn^2+^-release was measured by monitoring the Zn^2+^-PAR complex at 494 nm every 1 s for 30 min. The Zn^2+^ content of purified proteins by PAR assay was determined using a molar extinction coefficient of 85,000 M^-1^cm^-1^ at 494 nm for Zn^2+^-PAR complex.

### MALDI-TOF MS and LC-ESI MS/MS analysis

The analysis of protein oxidation after overexpression in *E*. *coli* was performed as previously described [17, 18]. Briefly, aliquots of *E*. *coli* cells (1.8 ml of culture of LE0001, LE0002, LE0008, LE0009, or LE0010) were either treated with 1 mM H_2_O_2_ (final concentrations) for 1 min or not. Cells harvested by centrifugation after the addition of 200 μM of trichloroacetic acid (TCA) were sonicated in 500 μl of 10% TCA. The pellets obtained by centrifugation were resuspended with 20 μl IA buffer (50 mM iodoacetamide, 0.5 M Tris pH 8.0, 5% glycerol, 100 mM NaCl, 1 mM EDTA, 2% SDS) and incubated for 1 h in the dark to alkylate free thiols. After separation on 13.3% Tris-Tricine SDS-PAGE and staining with Coomassie Brilliant Blue R-250, protein bands were cut and analyzed by MALDI-TOF MS using a Voyager-DE STR instrument (Applied Biosystems) after in-gel tryptic digestion. The sites of oxidation were identified by LC-MS/MS analyses using an Agilent nanoflow-1200 series HPLC system connected to a linear ion trap mass spectrometer (Thermo Scientific).

### Construction of deletion mutant, complementation, and reporter fusion strains

The *B*. *subtilis fur* deletion mutant strain (HBL100) was constructed using long-flanking homology PCR as described previously [19]. The *fur zur* double mutant strain (HBL112) was generated by transformation of HBL100 with *zur*::*tet* cassette, and the *perR fur zur* triple mutant strain (LB1066) was generated by transformation of HBL112 with *perR*::*cat* cassette.

For the expression of FLAG fusion proteins from their own promoter in *B*. *subtilis*, the PCR fragments containing ORF and about 200 bp upstream region (*bl00075*, *bl05249*, *bl03703*, *bl00690*, *bl00950*, *fur*_*BS*_, *zur*_*BS*_) were individually cloned into *Bam*HI and *Eag*I sites of pJL070. For the expression of FLAG fusion proteins from *xylA* promoter in *B*. *subtilis*, the pXT plasmid which can fuse a xylose-inducible promoter to the gene of interest was used. The PCR fragments containing ribosome binding sequence and *perR* ORF from pJL070 were cloned into *Bam*HI and *Eco*RI sties of pXT, generating pJL240. Then, the PCR fragments containing consensus ribosome binding sequence and ORF (*perR*_*BS*_, *fur*_*BS*_, *zur*_*BS*_, *bl00690*, and *bl00950*) were each cloned into *Bam*HI and *Eag*I sites of pJL240. For the expression of PerR_BS_-FLAG, BL00690-FLAG, and BL00950-FLAG from *B*. *subtilis perR* promoter in *B*. *subtilis* (for the construction of LB2128, LB4034, and LB4106 strains), *Nde*I site was introduced at the beginning of *perR* ORF in pJL070 by QuikChange site-directed mutagenesis (Stratagene) generating pJL448. Then, the PCR amplified *bl00690* and *bl00950* ORFs were each cloned into *Nde*I and *Eag*I sites of pJL448. The *ScaI* digest of each plasmid was introduced to the corresponding *B*. *subtilis* strain to generate a transformant containing FLAG-fused gene in the *amyE* (pJL070-derived plasmids) or *thrC* (pJL240-derived plasmids) locus. The reporter fusion strains were constructed by transduction with SPβ phages, and β-galactosidase assays were performed, as described previously [15].

## Results

### Identification of five Fur family proteins in *B*. *licheniformis*

**Fig 1:**
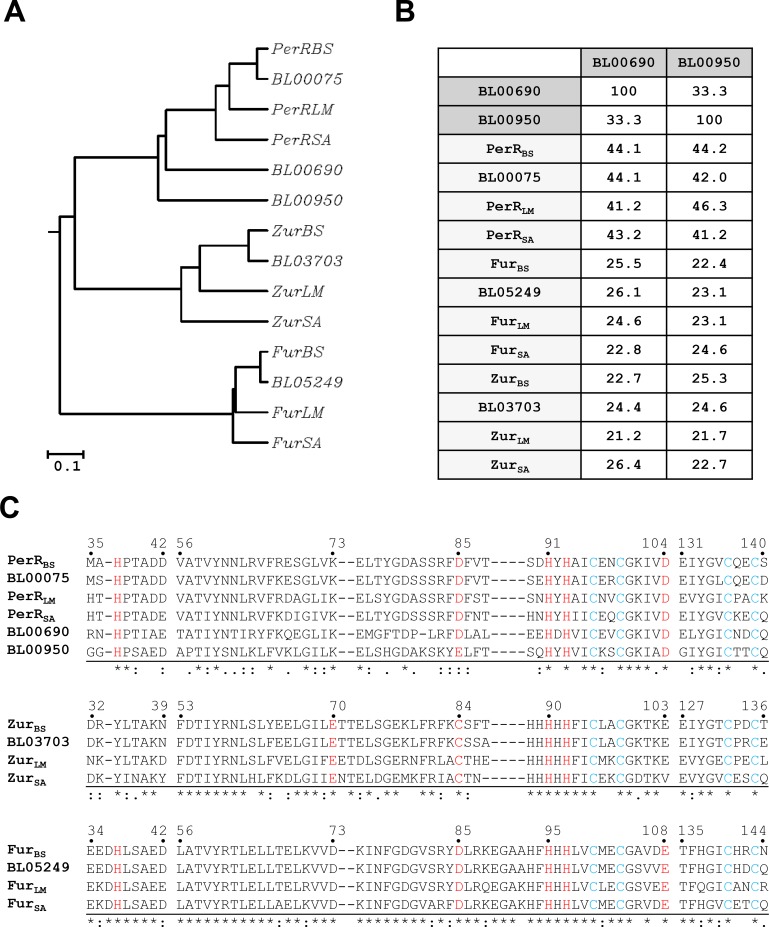
Sequence analysis of five Fur family proteins from *B*. *licheniformis*. (A) Phylogenetic tree of Fur-like proteins. The tree was constructed based on a multiple sequence alignment of Fur-like proteins from *Bacillus subtilis* (BS), *Bacillus licheniformis* (BL), *Listeria monocytogenes* (LM), and *Staphylococcus aureus* (SA), using CLUSTALW. Note that BL00690 and BL00950 cluster with PerR proteins. The scale bar represents an estimated distance of 0.1 amino acid substitution/site. (B) Amino acid identity matrix for BL00690 and BL00950. The amino acid sequences of BL00690 and BL00950 were compared with other Fur family proteins. The sequence identity values were shown as %. (C) Sequence alignment of Fur family proteins. The predicted structural Zn^2+^-binding site (blue) and regulatory metal binding site (red) are highly conserved in all five Fur-like proteins from *B*. *licheniformis*. The numbers above each sequence alignment group correspond to the sequence numbers of PerR_BS_, Zur_BS_, and Fur_BS_, respectively.

Many Gram positive bacteria such as *B*. *subtilis*, *L*. *monocytogenes* and *S*. *aureus* encode three Fur family proteins: Fur, Zur, and PerR [20–22]. Interestingly, the BLAST homology searches of the *B*. *licheniformis* ATCC14580 genome sequence [13] with each one of the *B*. *subtilis* Fur family proteins revealed the presence of five putative genes encoding Fur family proteins. BL00075, BL03703, and BL05249 of *B*. *licheniformis* show the highest similarity to PerR_BS_, Zur_BS_, and Fur_BS_ from *B*. *subtilis*, respectively, and all these proteins cluster with their homologues from *L*. *monocytogenes* and *S*. *aureus* as well as *B*. *subtilis* (Fig 1A). Although the sequence identity between BL00690 and BL00950 is not high (33%), both proteins cluster with PerR proteins with sequence identities ranging between 41 and 44% for BL00690 and between 41 and 46% for BL00950 (Fig 1B). In comparison, BL00690 and BL00950 exhibit sequence identities of ~25% to Fur and Zur proteins (Fig 1B), which are comparable to those between PerR and Fur or between PerR and Zur [1, 2, 22, 23].

As shown in Fig 1C, all the five Fur family proteins from *B*. *licheniformis* retain four highly conserved Cys residues corresponding to Cys96, Cys99, Cys136, and Cys139 of *B*. *subtilis* PerR. These four Cys residues arranged in two CXXC motifs are involved in high affinity structural Zn^2+^-binding in most Fur family proteins including PerR_BS_ [9, 15]. In addition to this structural Zn^2+^-binding site, Fur family proteins also have a regulatory metal binding site. For PerR_BS_, this site is composed of His37, Asp85, His91, His93 and Asp104 [9, 11]. These five residues are conserved in BL00690 as well as PerR proteins including BL00075. Although Fur proteins and BL00950 also have conserved N-donor ligands (corresponding to His37, His91, and His93 of PerR_BS_), these proteins have a Glu residue in place of Asp104 (for Fur proteins) or Asp85 (for BL00950) as an O-donor ligand. Zur proteins are known to use S-donor ligand corresponding to Cys84 of Zur_BS_ instead of O-donor ligand corresponding to Asp85 of PerR_BS_ for regulatory Zn^2+^-binding, and do not have a conserved N-donor ligand corresponding to His37 of PerR_BS_ [3, 5, 7]. Based on their repressor activities as described below as well as their sequence similarity and conserved amino acid residues involved in putative structural and regulatory metal binding, we functionally annotate BL00075, BL03703, and BL05249 as PerR_BL_, Zur_BL_, and Fur_BL_, respectively. And, the new Fur homologues, BL00690 and BL00950, were annotated as PerR2 and PerR3, respectively, based on their sequence similarity to the PerR proteins and their ability to sense peroxide by histidine oxidation as described below.

### All the five Fur family proteins from *B*. *licheniformis* contain structural Zn^2+^

**Fig 2:**
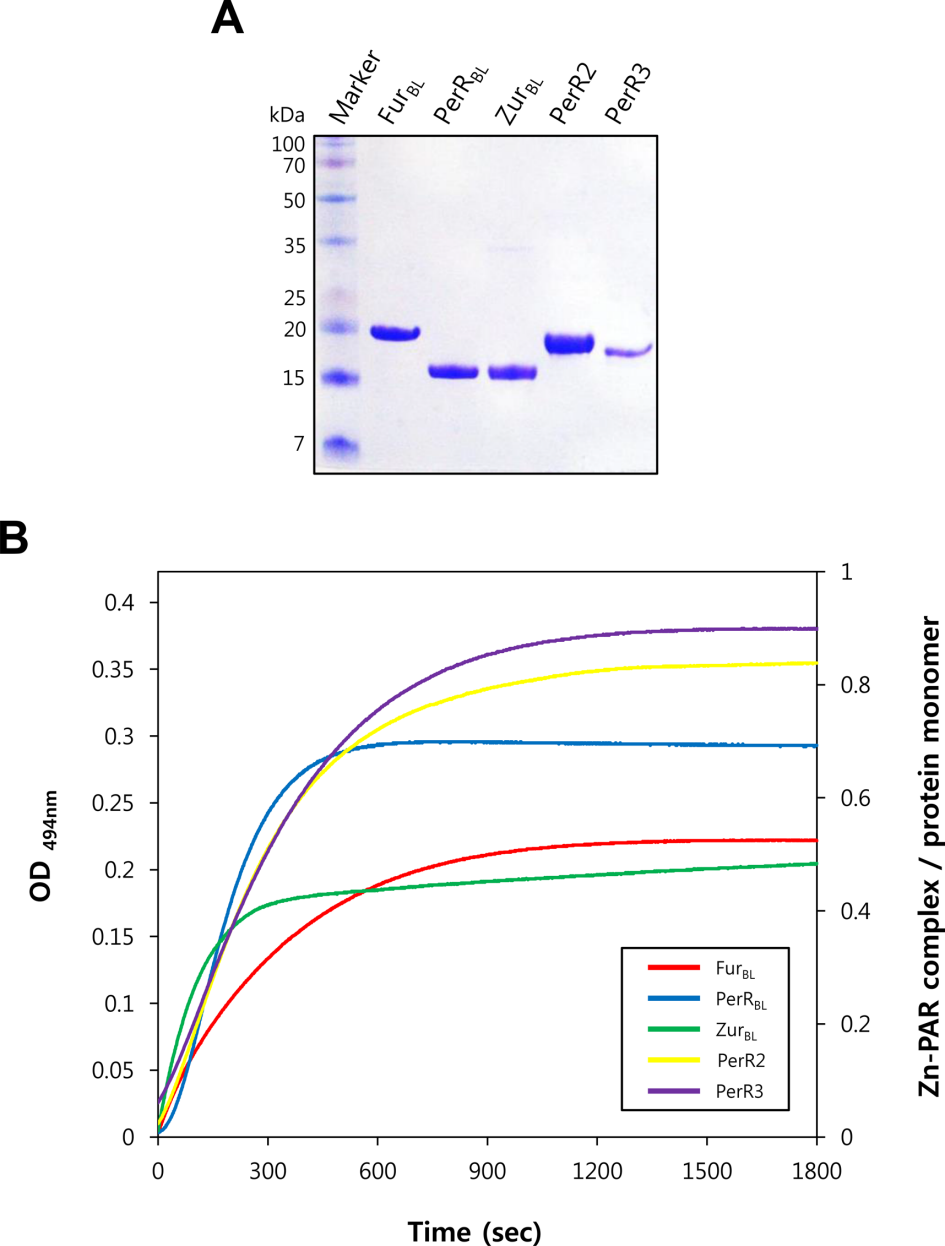
Zn^2+^-contents of Fur family proteins from *B*. *licheniformis*. (A) Purified Fur family proteins from *B*. *licheniformis*. *B*. *licheniformis* Fur-like proteins were purified after overexpression in *E*. *coli*, and analyzed by SDS-PAGE after alkylation by iodoacetamide. (B) H_2_O_2_-dependent Zn^2+^-release. Release of Zn^2+^ from proteins (5 μM) was measured by monitoring Zn^2+^-PAR complex at 494 nm every 1 sec for 30 min after treatment of 0, 1, 10, and 100 mM H_2_O_2_. Data for experiments with 100 mM H_2_O_2_ are only shown for clarity. The Zn^2+^-content of proteins was calculated using a molar extinction coefficient of 85,000 M^-1^cm^-1^ at 494 nm for Zn^2+^-PAR complex.

The sequence analysis indicates that all the Fur family proteins from *B*. *licheniformis* have conserved cysteine residues putatively involved in structural Zn^2+^-binding. To investigate the involvement of cysteine residues in Zn^2+^ coordination, we purified all the five Fur family proteins after overexpression in *E*. *coli* (Fig 2A), and measured Zn^2+^-release from each protein upon H_2_O_2_ treatment by monitoring the formation of PAR-Zn^2+^ complex (Fig 2B) as described previously [15, 17]. Interestingly, unlike other Fur family proteins PerR3 did not bind to heparin-Sepharose (which is widely used for the purification of DNA-binding proteins). Furthermore, PerR3 was purified as monomeric protein by a gel filtration chromatography, whereas the other four Fur family proteins were purified as dimeric proteins (see Materials and Methods).

PAR-Zn^2+^ complex formation was not detected for 30 min without H_2_O_2_ treatment, and the rate of Zn^2+^-release was dependent on added H_2_O_2_ concentrations (Fig 2B). These results indicate that all the Fur family proteins from *B*. *licheniformis*, including PerR3 purified as monomers, have stably bound Zn^2+^ which cannot easily be removed by high affinity Zn^2+^-chelator PAR (*K*_app_ ~ 10^13^ M^-2^ for PAR_2_-Zn^2+^ complex, [24]) in the absence of H_2_O_2_. Furthermore, the dependence of Zn^2+^-release on H_2_O_2_ strongly suggests that Zn^2+^ is coordinated by conserved cysteine residues as observed with PerR proteins [15, 17]. The second-order rate constants of Zn^2+^ release by H_2_O_2_ were determined to be ~0.03 M^-1^s^-1^ for PerR2, PerR3, and Fur_BL_, ~0.04 M^-1^s^-1^ for PerR_BL_, and ~0.01 M^-1^s^-1^ for Zur_BL_. The slow rates of H_2_O_2_-mediated Zn^2+^ release for Fur family proteins from *B*. *licheniformis*, which are comparable to those observed with *B*. *subtilis* and *S*. *aureus* PerR proteins (~0.05 M^-1^s^-1^) [15, 17], suggest that the Zn^2+^ sites play a structural rather than a H_2_O_2_ sensing role. The Zn^2+^ contents of the purified proteins per monomer were determined to be ~0.8 for PerR2, ~0.9 for PerR3, ~0.5 for Fur_BL_, ~0.7 for PerR_BL_, and ~0.5 for Zur_BL_. The retention of ~0.5–0.9 Zn^2+^ per monomer, despite the use of strong metal chelator EDTA during protein purification (see Materials and Methods), also supports the notion that all the Fur family proteins from *B*. *licheniformis* have a structural Zn^2+^ site. Altogether, these data indicate that all the five Fur family proteins from *B*. *licheniformis* contain a structural Zn^2+^ presumably coordinated by conserved cysteine residues like many other Fur proteins.

### PerR2 (BL00690) and PerR3 (BL00950) as well as PerR_BL_ can sense H_2_O_2_ by protein oxidation

**Fig 3:**
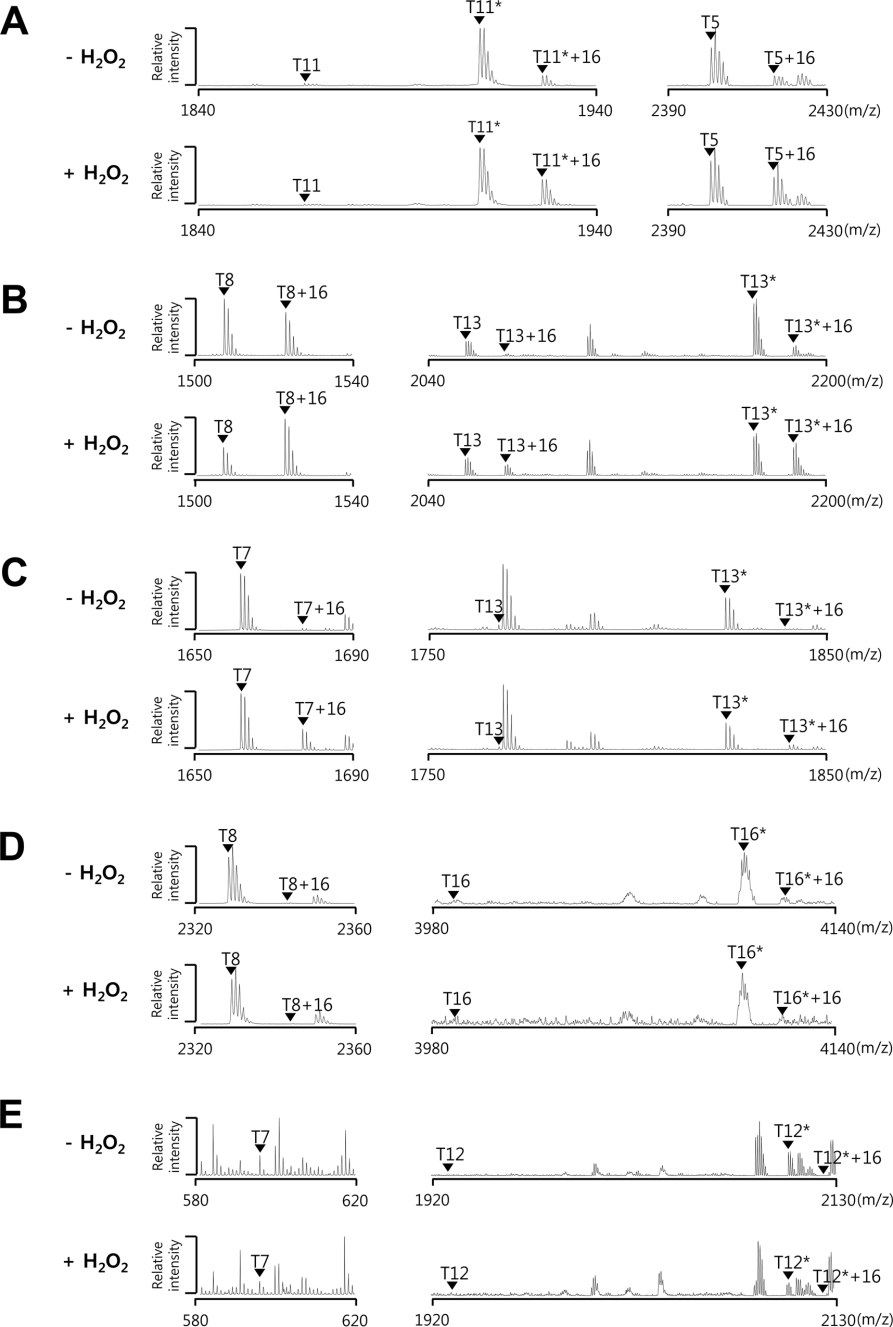
H_2_O_2_-dependent oxidation of Fur family proteins from *B*. *licheniformis*. Oxidation of PerR_BL_ (A), PerR2 (B), PerR3 (C), Fur_BL_ (D), and Zur_BL_ (E) before and after H_2_O_2_ treatment. *E*. *coli* cells expressing PerR_BL_ (LE0008), PerR2 (LE0002), PerR3 (LE0001), Fur_BL_ (LE0009), or Zur_BL_ (LE0010), were treated with or without 1 mM H_2_O_2_ for 1 min. Oxidation status of proteins was analyzed by MALDI-TOF MS after SDS-PAGE fractionation and in-gel tryptic digestion. Asterisks represent peptides containing one (PerR_BL_ and PerR3), two (PerR2 and Fur_BL_), or three (Zur_BL_) carboxyamidomethylated cysteine residues.

Previously we have shown that the oxidation of PerR proteins can be easily and efficiently evaluated using *E*. *coli* system [17, 18]. To investigate the oxidation of Fur family proteins from *B*. *licheniformis*, we analyzed protein oxidation by MALDI-TOF MS after overexpression in *E*. *coli* with or without H_2_O_2_ treatment (Fig 3) as described previously [17, 18]. As noted for PerR_BS_, PerR_BL_ showed H_2_O_2_-dependent oxidation at two tryptic peptides, T5 (His25 to Lys45, m/z = 2401.19) containing His37 and T11* (Phe84 to Arg98, m/z = 1910.85) containing His91, befitting its role as PerR (Fig 3A). In contrast, Fur_BL_ and Zur_BL_ displayed no detectable changes in tryptic peptide peaks after H_2_O_2_ treatment (Fig 3D and 3E). Interestingly, PerR2 exhibited significant degree of oxidation at T8 peptide (Asn38 to Arg50, m/z = 1506.80) containing His39 (corresponding to His37 in PerR_BS_) even without H_2_O_2_ treatment, and further oxidation at T8 peptide and T13* peptide (Phe85 to Lys102, m/z = 2170.99) containing His92 (corresponding to His91 in PerR_BS_) after H_2_O_2_ treatment (Fig 3B). PerR3 also displayed H_2_O_2_-dependent oxidation, although less when compared with PerR_BL_ and PerR2, at T7 peptide (Thr27 to Arg42, m/z = 1660.84) containing His34 (corresponding to His37 in PerR_BS_) and T13* peptide (Tyr77 to Lys90, m/z = 1824.87) containing His84 (corresponding to His91 in PerR_BS_) (Fig 3C). As expected, the sites of oxidation responsible for the 16 Da mass increase were mapped to be His37 and His91 for PerR_BL_, His39 and His92 for PerR2, and His34 for PerR3 (S1–S5 Figs). The site of oxidation for T13*+16 from PerR3 could not be exactly mapped partially due to the weak signal intensity. The presence of significantly oxidized T8 peptide (T8+16) from PerR2 as compared to that (T5+16) from PerR_BL_ in the absence of H_2_O_2_ treatment suggests that PerR2 is more sensitive than PerR_BL_ to oxidation by H_2_O_2_ encountered during aerobic growth of *E*. *coli* [17]. In addition, no detectable oxidation without external H_2_O_2_ treatment and the inefficient oxidation by H_2_O_2_ treatment for PerR3 suggest that PerR3 is less sensitive to oxidation by H_2_O_2_ than PerR_BL_ or PerR2.

All the peptides (T11* peptide of PerR_BL_, T13* peptide of PerR2, and T13* peptide of PerR3) containing putative Zn^2+^-binding motif CXXC motif (corresponding to C_96_XXC_99_ in PerR_BS_) were detected in their fully alkylated form (Fig 3, S2 and S4 Figs). Note that the small amount of T13 peptide of PerR2, which is detected without alkylation even in the absence of H_2_O_2_ treatment, underwent no further oxidation after H_2_O_2_ treatment (Fig 3B). This observation that the cysteine residues are refractory to oxidation by H_2_O_2_ treatment is consistent with the idea that these cysteine residues are involved in structural Zn^2+^-binding. All these data together suggest that PerR2 and PerR3 as well as PerR_BL_ can sense H_2_O_2_ with differential sensitivity, by histidine oxidation but not by cysteine oxidation.

### Functional annotation of PerR_BL_ (BL00075) as PerR, Fur_BL_ (BL05249) as Fur, and Zur_BL_ (BL03703) as Zur

**Fig 4:**
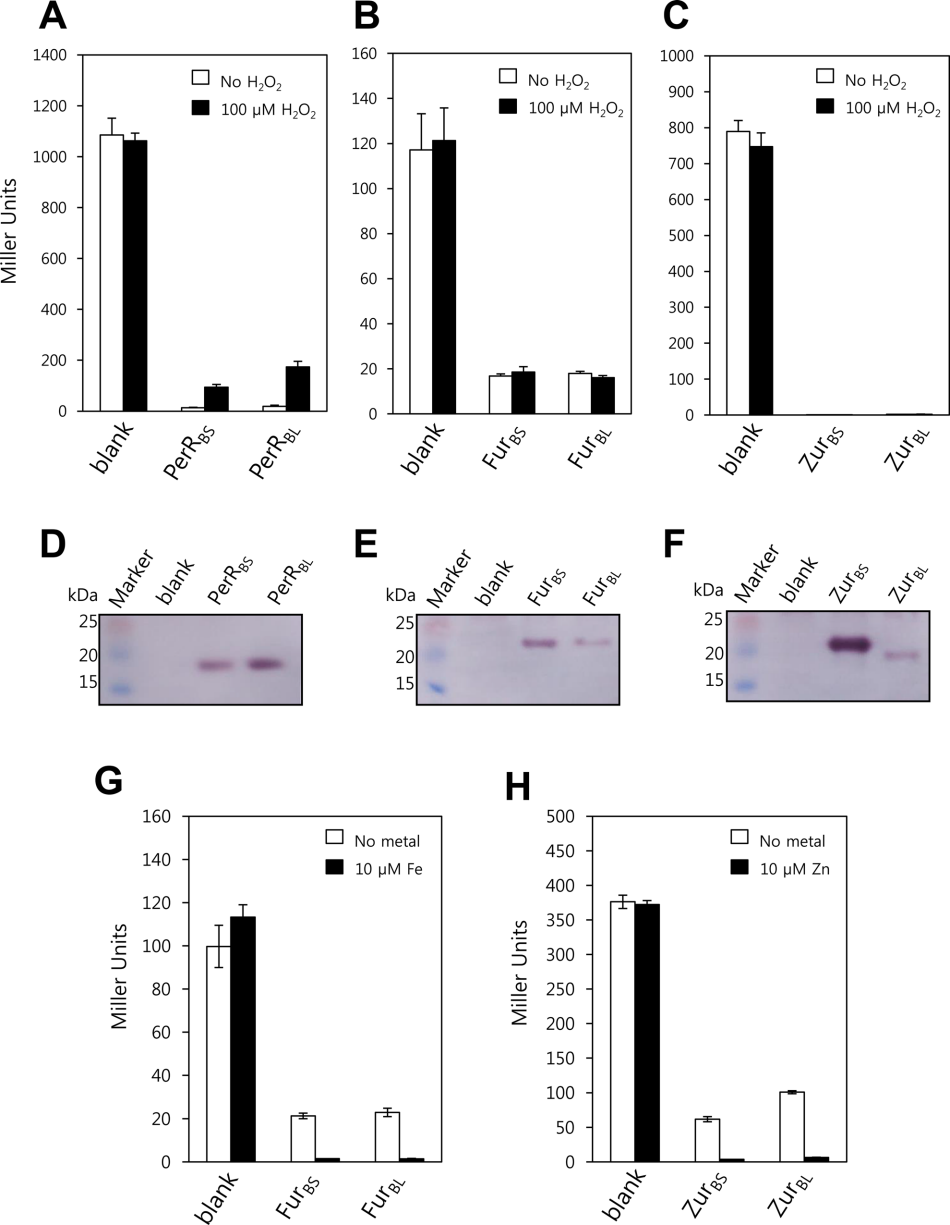
***In vivo* repressor activities of PerR**_**BL**_**, Fur**_**BL**_**, and Zur**_**BL**_ (A) Repressor activities of PerR_BS_ and PerR_BL_ for *P*_*mrgA*_*-lacZ* reporter fusion. *B*. *subtilis* cells expressing no PerR orthologue (LB1532), PerR_BS_-FLAG (HB9738), or PerR_BL_-FLAG (LB1023) were treated without or with 100 μM H_2_O_2_ for 30 min, and β-galactosidase activities were measured using *P*_*mrgA*_*-lacZ* reporter fusion. (B) Repressor activities of Fur_BS_ and Fur_BL_ for *P*_*feuA*_*-lacZ* reporter fusion. *B*. *subtilis* cells expressing no Fur orthologue (LB1040), Fur_BS_-FLAG (LB1041), or Fur_BL_-FLAG (LB1042) were treated without or with 100 μM H_2_O_2_ for 30 min, and β-galactosidase activities were measured using *P*_*feuA*_*-lacZ* reporter fusion. (C) Repressor activities of Zur_BS_ and Zur_BL_ for *P*_*yciC*_*-lacZ* reporter fusion. *B*. *subtilis* cells expressing no Zur orthologue (LB1034), Zur_BS_-FLAG (LB1035), or Zur_BL_-FLAG (LB1036) were treated without or with 100 μM H_2_O_2_ for 30 min, and β-galactosidase activities were measured using *P*_*yciC*_*-lacZ* reporter fusion. (D-F) Western blot analyses of FLAG-fused PerR orthologues (D), Fur orthologues (E), and Zur orthologues (F). The FLAG-fused proteins were probed by anti-FLAG antibody. (G) Fe-dependent repressor activities of Fur_BS_ and Fur_BL_ for *P*_*feuA*_*-lacZ* reporter fusion. *B*. *subtilis* cells expressing no Fur orthologue (LB1040), Fur_BS_-FLAG (LB1041), or Fur_BL_-FLAG (LB1042) were grown in MLMM supplemented with or without 10 μM FeSO_4_, and β-galactosidase activities were measured using *P*_*feuA*_*-lacZ* reporter fusion. (H) Zn-dependent repressor activities of Zur_BS_ and Zur_BL_ for *P*_*yciC*_*-lacZ* reporter fusion. *B*. *subtilis* cells expressing no Zur orthologue (LB1034), Zur_BS_-FLAG (LB1035), or Zur_BL_-FLAG (LB1036) were grown in MLMM supplemented with or without 10 μM ZnCl_2_, and β-galactosidase activities were measured using *P*_*yciC*_*-lacZ* reporter fusion.

The function of PerR, Zur, and Fur have intensively been studied both structurally and molecular genetically in *B*. *subtilis* [11, 25–28], a close relative of *B*. *licheniformis*. Thus, *B*. *subtilis* provides an excellent model system for the characterization of Fur family proteins from *B*. *licheniformis*. To investigate whether PerR_BL_ (BL00075) functions as PerR, Fur_BL_ (BL05249) as Fur, and Zur_BL_ (BL03703) as Zur, heterologous complementation studies were performed using *B*. *subtilis*. For this, PerR_BL_-FLAG, Fur_BL_-FLAG, or Zur_BL_-FLAG was expressed from its own promoter (with ~200 nucleotide sequence upstream of ORF) in a *B*. *subtilis* strain lacking a functional *perR*, *zur*, or *fur* gene, respectively (Fig 4). Since the FLAG epitope-tagged *B*. *subtilis* Fur family proteins are fully functional and the epitope tag provides a convenient means of monitoring protein levels *in vivo*, C-terminal FLAG-tagged proteins were used for activity analyses *in vivo* [11, 17, 26, 29].

The repressor activity of PerR_BL_-FLAG was monitored using a *B*. *subtilis mrgA* promoter-*lacZ* reporter fusion (*P*_*mrgA*_-*lacZ*) which is under the control of PerR_BS_. As reported previously [11], the *P*_*mrgA*_-*lacZ* was repressed in cells expressing PerR_BS_-FLAG but derepressed in the *perR* null mutant cells. The *P*_*mrgA*_-*lacZ* was also fully repressed by PerR_BL_-FLAG, and the repression was relieved upon H_2_O_2_ treatment as observed with PerR_BS_-FLAG (Fig 4A). Fur_BL_ showed a full repressor activity for Fur_BS_-regulated *feuA* promoter-*lacZ* reporter fusion (*P*_*feuA*_-*lacZ*) (Fig 4B). Zur_BL_-FLAG exhibited a full repressor activity for *B*. *subtilis yciC* promoter-*lacZ* reporter fusion (*P*_*yciC*_-*lacZ*) which is under the control of Zur_BS_, despite the lower levels of expression when compared to Zur_BS_-FLAG (Fig 4C). We also examined the metal-dependent repressor activities of Fur_BL_ and Zur_BL_ using a metal-limited minimal medium (MLMM). As expected, Fur_BL_ fully repressed the *P*_*feuA*_-*lacZ* in the presence of Fe like Fur_BS_, and Zur_BL_ fully repressed the *P*_*yciC*_-*lacZ* in the presence of Zn like Zur_BS_ (Fig 4G and 4H).

These results imply that PerR_BL_ (BL00075), Fur_BL_ (BL05249), and Zur_BL_ (BL03703) may function as PerR, Fur, and Zur, respectively, in *B*. *licheniformis*, and that each protein can be expressed from its own promoter located in ~200 nucleotide sequence upstream of each ORF.

### PerR2 (BL00690), but not PerR3 (BL00950), has a PerR-like repressor activity

**Fig 5:**
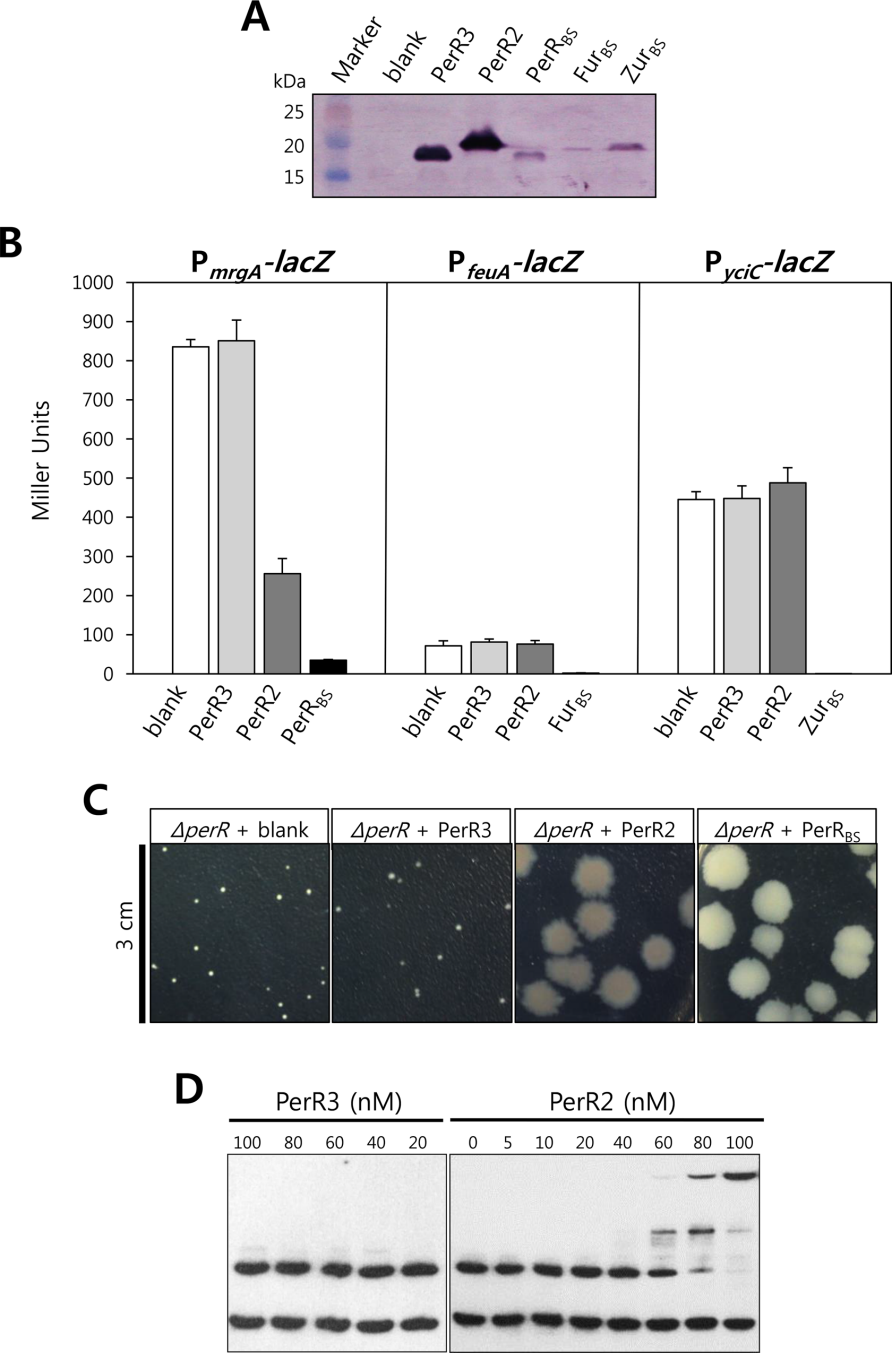
Repressor activity of PerR2. (A) Western blot analysis of FLAG-fused Fur family proteins expressed from *xylA* promoter. *B*. *subtilis perR fur zur* triple mutant cells expressing no Fur family protein (LB1227), PerR3 (LB1287), PerR2 (LB1288), PerR_BS_ (LB1490), Fur_BS_ (LB1491), and Zur_BS_(LB1493) were used. (B) Repressor activities of PerR2 and PerR3. Repressor activities of PerR3 and PerR2 were measured using *P*_*mrgA*_*-lacZ*, *P*_*feuA*_*-lacZ*, and *P*_*yciC*_*-lacZ* reporter fusions. As a control, repressor activities of PerR_BS_, Fur_BS_, and Zur_BS_ were also measured using *P*_*mrgA*_*-lacZ*, *P*_*feuA*_*-lacZ*, and *P*_*yciC*_*-lacZ* reporter fusions, respectively. The reporter fusion strains were constructed from the strains used in Fig 5A (Table 1). (C) Effects of PerR2 and PerR3 on the growth of the *B*. *subtilis perR* deletion mutant strain. The *B*. *subtilis perR* deletion mutant cells expressing no PerR (LB1010), PerR_BS_ (LB2128) PerR2 (LB4034), or PerR3 (LB4106) were grown on LB agar plate for 1 day. (D) DNA binding activities of PerR2 and PerR3. DNA binding activities of PerR2 and PerR3 were measured by EMSA in the presence of 100 μM MnCl_2_.

PerR2-FLAG and PerR3-FLAG could not be expressed with ~200 nucleotide sequence upstream of their ORFs, thus it is likely that the genes encoding these proteins do not have their own promoters. To express PerR2-FLAG and PerR3-FLAG and investigate the roles of these proteins *in vivo*, we used the pXT system which fuses a xylose-inducible promoter to the gene of interest, and a triple mutant *B*. *subtilis* strain which lacks all of the three *fur* family genes (Fig 5). Despite the use of same *xylA* promoter along with the consensus ribosome binding site, the expression levels of Fur family proteins were not identical possibly by differences in mRNA and/or protein stability (Fig 5A). However, as observed with single mutant background with their own promoters (Fig 4), PerR_BS_-FLAG, Fur_BS_-FLAG, and Zur_BS_-FLAG expressed from *xylA* promoter fully repressed the *P*_*mrgA*_-*lacZ*, *P*_*feuA*_-*lacZ*, and *P*_*yciC*_-*lacZ*, respectively (Fig 5B). Although PerR3-FLAG was highly expressed under the control of *xylA* promoter, PerR3-FLAG showed no repressor activity for PerR-regulated reporter fusion as well as for Fur- and Zur-regulated reporter fusions. Interestingly, PerR2-FLAG exhibited repressor activity for the PerR-regulated *P*_*mrgA*_-*lacZ*, but no repressor activity for the Fur-regulated *P*_*feuA*_-*lacZ* nor the Zur-regulated *P*_*yciC*_-*lacZ*. This specific repressor activity of PerR2 for the known PerR-regulated promoters, along with its H_2_O_2_-dependent histidine oxidation, suggest that PerR2 may act as a second PerR in *B*. *licheniformis*.

It is known that a *B*. *subtilis perR* null mutant strain grows very poorly in nonstressed conditions due to Fe deficiency resulting from elevated levels of Fur_BS_ and KatA [19]. To examine whether PerR2 can complement the *perR* null mutant strain and rescue the small colony phenotype, complementation experiments were performed (Fig 5C). As expected, the *perR* null mutant strain expressing PerR_BS_-FLAG showed a wild-type like colony phenotype. The *perR* null mutant strain expressing PerR2 also exhibited significantly increased colony size, indicating that PerR2 can rescue the Fe-deficiency presumably by reducing the levels of KatA and/or Fur_BS_. In contrast, the *perR* null mutant strain expressing PerR3 still exhibited the small colony phenotype (Fig 5C) consistent with the lack of repressor activity for the PerR-regulated gene (Fig 5B).

To investigate the interaction of PerR2 with DNA, we performed electrophoretic mobility shift assays using the *B*. *subtilis mrgA* promoter regions as probe. As shown in Fig 5D, PerR2 specifically shifted the DNA fragment containing PerR box but not the DNA fragment lacking PerR box. This result indicates that the repressor activity of PerR2 observed with the PerR-regulated promoter fusion is due to direct interaction of PerR2 with PerR box. However, it should be noted that the apparent *K*_*d*_ value of PerR2 for DNA binding was measured to be ~ 70 nM. This rather weak DNA binding activity of PerR2, as compared to that of PerR_BS_ (*K*_*d*_ ~ 10 nM) [29], is likely to reflect the higher oxidation (inactivation) levels of PerR2 as shown in Fig 3B. In contrast, consistent with the lack of repressor activity for the PerR regulated promoter fusion, PerR3 showed no DNA binding activity (Fig 5D).

## Discussions

Proteins with Fur-like domain architecture are widespread in prokaryotes with ~20,000 homologues in EMBL-EBI InterPro database (IPR002481). Depending on signals they respond, Fur family proteins are classified as Fur (Fe), Zur (Zn), Mur (Mn), Nur (Ni), PerR (peroxide), and Irr (heme) [1, 2]. Among these, Fur is the most ubiquitous, and Zur, albeit not as ubiquitous as Fur, is also widespread in Gram negative and Gram positive bacteria. In contrast, PerR is mainly found in Gram positive bacteria as a functional substitute for OxyR, although it is also found in some Gram negative bacteria, and, in some cases, coexists with OxyR [1, 10]. Mur and Irr have been found in some α-proteobacteria including *Rhizobiales* and *Rhodobacterales* [30], and Nur has been only found in *Streptomyces* genus [31]. Although four Fur paralogues (Fur orthologue FurA, PerR orthologue CatR, Zur, and Nur) have been found and characterized in *S*. *coelicolor* [3, 31–33], many bacteria contains up to three Fur family proteins, usually two or three. For example, Gram negative bacteria *E*. *coli* and *V*. *cholerae* contain two (Fur and Zur) [4, 7], and Gram positive bacteria *B*. *subtilis* and *S*. *aureus* contain three (Fur, Zur, and PerR) [20, 22]. In this study, we found that *B*. *licheniformis*, a close relative of *B*. *subtilis*, contains five Fur family proteins. Like many other Fur family proteins, all these proteins retain a tightly bound Zn^2+^ presumably coordinated by highly conserved cysteine residues. Three of them were identified as Fur, Zur, and PerR orthologues of *B*. *subtilis* based on their repressor activity. The other two were identified as PerR-like proteins based on their sequence similarity to PerR proteins and their H_2_O_2_-dependent oxidation of histidine residues.

The H_2_O_2_-sensing mechanism of PerR has only been extensively studied in *B*. *subtilis* and *S*. *aureus*, despite its wide distribution in most Gram positive bacteria and in some Gram negative bacteria [10, 11, 17, 25]. Unlike OxyR which utilizes the oxidation of cysteine thiol, PerR uses a distinct Fe-dependent histidine oxidation mechanism for H_2_O_2_ sensing, where H_2_O_2_ oxidizes the histidine ligands of the Fe^2+^ at the regulatory site to 2-oxo-histidine. Our results indicate that PerR_BL_ also uses a histidine oxidation mechanism for H_2_O_2_ sensing. Furthermore, we found that H_2_O_2_ can also oxidize the two other PerR-like proteins, PerR2 and PerR3, but not Fur_BL_ and Zur_BL_. MALDI-TOF MS and ESI-MS/MS analyses of the tryptic peptides, along with sequence analyses, of PerR2 and PerR3 indicate that the oxidation events occur at histidine residues rather than cysteine residues. Despite the high similarity between the regulatory metal binding sites of PerR and Fur, Fur does not react with H_2_O_2_ under conditions where PerR does [11]. Recently, it has been suggested that O-donor ligand corresponding to Asp104 of PerR_BS_ or Glu108 of Fur_BS_ is the key residue which determines the accessibility of H_2_O_2_ to Fe^2+^-coordination site [12]. It is noteworthy that PerR2 and PerR3, as well as other PerR proteins, also contain a conserved Asp at this position, whereas Fur proteins have a Glu (Fig 1C).

Despite the presence of *bona fide* PerR_BL_, PerR2 also showed specific repressor activity on the representative PerR-regulated gene but not on Fur- or Zur-regulated gene, and the *perR* null mutant small colony phenotype could be rescued by PerR2 (Fig 5). Thus, it is reasonable to speculate that PerR regulon in *B*. *licheniformis* is under the control of both PerR_BL_ and PerR2. In the simplest scenario, the two proteins would exert influence on the PerR regulon genes simultaneously. Alternatively, each protein may regulate genes under different conditions. The higher sensitivity of PerR2 than PerR_BL_ seems to suggest the differential role of these proteins under different oxidation conditions.

Unlike PerR2, no repressor activity of PerR3 was observed for any genes under the control of PerR, Fur, and Zur using *B*. *subtilis* reporter fusion assays. And, PerR3 was purified as monomer after overexpression in *E*. *coli*, whereas all the other four Fur family proteins from *B*. *licheniformis* were purified as dimer. Considering that all the biochemically characterized Fur family proteins are dimeric DNA binding proteins, PerR3 may not be a canonical Fur family protein. However, the oxidation of PerR3 by H_2_O_2_, albeit less sensitive as compared to PerR_BL_ or PerR2, suggests that PerR3 may play a role as a H_2_O_2_ sensor in *B*. *licheniformis*. It has been previously reported that the transcription of *perR3* (*bl00950*, *bli04114)* is massively induced after H_2_O_2_ treatment [34]. Interestingly, the genes encoding for PerR3 and BL00949 (BLi04115, putative ferrochelatase) are located directly downstream of *katA* gene (*bl00951*, *bli04113*). Furthermore, in contrast to *B*. *subtilis katA* gene which is monocistronically transcribed under the control of PerR_BS_, *B*. *licheniformis katA* gene is cotranscribed with *perR3* and *bl00949* after H_2_O_2_ treatment [34]. These imply that PerR3 may have some role especially under conditions of H_2_O_2_ stress.

In summary, we have shown that *B*. *licheniformis* contains a total of five Fur family proteins: two novel PerR-like proteins in addition to the canonical PerR, Fur, and Zur. The presence of two additional Fur family proteins in *B*. *licheniformis*, in contrast to its close relative *B*. *subtilis*, may indicate that the metal ion regulation and peroxide stress response under the control of Fur family proteins are far more complex than previously reported for *B*. *subtilis*. Further study is required to identify distinct roles of PerR2 and PerR3 along with their relevance to other Fur family proteins in *B*. *licheniformis*.

## Supporting Information

S1 Fig Identification of His37 as an oxidation site in T5 peptide (His25 to Lys45) from PerR_BL_ by ESI MS/MS analysis. (A) Predicted m/z values of b- and y-ions of unmodified T5 peptide of PerR_BL_. His37 is shown in red. (B) Tandem MS spectrum of T5 peptide. Triple charged precursor ion ([T5+3H]^3+^ = 801.85, shown in green) was analyzed by tandem MS. The b- and y-ions are shown in purple and blue, respectively. (C) Tandem MS spectrum of T5+16 peptide. Triple charged precursor ion ([T5+16+3H]^3+^ = 806.82, shown in green) was analyzed by tandem MS. The b- and y-ions are shown in purple and blue, respectively. The y-ions not containing His37 (y4-y8) appear at the predicted m/z values, whereas the subsequent y-ions containing His37 (y9-y20) have a +16 Da mass shift. Note that almost all the y9- and y10-ions (containing His37 but not Met35) have a +16 Da mass shift. The b-ions not containing His37 (b5-b12) appear at the predicted m/z values, whereas the subsequent b-ions containing His37 (b13-b20) have a +16 Da mass shift. Note that almost all the b10- and b11-ions (containing Met35 but not His37) appear at the predicted m/z values. Taken together, these data indicate that most of the oxidation in T5+16 peptide occurred at His37 rather than Met35.
(TIF)

S2 Fig Identification of His91 as an oxidation site in T11* peptide (Phe84 to Arg98) from PerR_BL_ by ESI MS/MS analysis. (A) Predicted m/z values of b- and y-ions of T11* peptide (containing carboxyamidomethylated Cys96 residue) of PerR_BL_. His91 is shown in red. (B) Tandem MS spectrum of T11* peptide. Double charged precursor ion ([T11*+2H]^2+^ = 956.06, shown in green) was analyzed by tandem MS. The b- and y-ions are shown in purple and blue, respectively. (C) Tandem MS spectrum of T11*+16 peptide. Double charged precursor ion ([T11*+16+2H]^2+^ = 964.42, shown in green) was analyzed by tandem MS. The b- and y-ions are shown in purple and blue, respectively. The y-ions not containing His91 (y3-y7) appear at the predicted m/z values, whereas the subsequent y-ions containing His91 (y8-y14) have a +16 Da mass shift. The b-ions not containing His91 (b3-b7) appear at the predicted m/z values, whereas the subsequent b-ions containing His91 (b8-b14) have a +16 Da mass shift. These data indicate that the oxidation in T11*+16 peptide occurred at His91.
(TIF)

S3 Fig Identification of His39 as an oxidation site in T8 peptide (Asn38 to Arg50) from PerR2 by ESI MS/MS analysis. (A) Predicted m/z values of b- and y-ions of T8 peptide of PerR2. His39 is shown in red. (B) Tandem MS spectrum of T8 peptide. Triple charged precursor ion ([T8+3H]^3+^ = 503.20, shown in green) was analyzed by tandem MS. The b- and y-ions are shown in purple and blue, respectively. (C) Tandem MS spectrum of T8+16 peptide. Triple charged precursor ion ([T8+16+3H]^3+^ = 509.16, shown in green) was analyzed by tandem MS. The b- and y-ions are shown in purple and blue, respectively. The y-ions not containing His39 (y1-y11) appear at the predicted m/z values, whereas the subsequent y-ions containing His39 (y12-y13) have a +16 Da mass shift. The b-ions containing His39 (b2-b13) have a +16 Da mass shift. These data indicate that the oxidation in T8+16 peptide occurred at His39.
(TIF)

S4 Fig Identification of His92 as an oxidation site in T13* peptide (Phe85 to Lys102) from PerR2 by ESI MS/MS analysis. (A) Predicted m/z values of b- and y-ions of T13* peptide (containing carboxyamidomethylated Cys97 and Cys100 residues) of PerR2. His92 is shown in red. (B) Tandem MS spectrum of T13* peptide. Triple charged precursor ion ([T13*+3H]^3+^ = 724.93, shown in green) was analyzed by tandem MS. The b- and y-ions are shown in purple and blue, respectively. (C) Tandem MS spectrum of T13*+16 peptide. Triple charged precursor ion ([T13*+16+3H]^3+^ = 730.05, shown in green) was analyzed by tandem MS. The b- and y-ions are shown in purple and blue, respectively. The y-ions not containing His92 (y3-y10) appear at the predicted m/z values, whereas the subsequent y-ions containing His92 (y11-y17) has a +16 Da mass shift. The b-ions not containing His92 (b3-b7) appear at the predicted m/z values, whereas the subsequent b-ions containing His92 (b8-b16) has a +16 Da mass shift. These data indicate that the oxidation in T8+16 peptide occurred at His92.
(TIF)

S5 Fig Identification of His34 as an oxidation site in T7 peptide (Thr27 to Arg42) from PerR3 by ESI MS/MS analysis. (A) Predicted m/z values of b- and y-ions of T7 peptide of PerR3. His34 is shown in red. (B) Tandem MS spectrum of T7 peptide. Double charged precursor ion ([T7+2H]^2+^ = 831.40, shown in green) was analyzed by tandem MS. The b- and y-ions are shown in purple and blue, respectively. (C) Tandem MS spectrum of T7+16 peptide. Double charged precursor ion ([T7+16+2H]^2+^ = 839.29, shown in green) was analyzed by tandem MS. The b- and y-ions are shown in purple and blue, respectively. The y-ions not containing His34 (y3-y8) appear at the predicted m/z values, whereas the subsequent y-ions containing His34 (y9-y15) has a +16 Da mass shift. The b-ions not containing His34 (b5-b7) appear at the predicted m/z values, whereas the subsequent b-ions containing His34 (b8-b16) has a +16 Da mass shift. These data indicate that the oxidation in T7+16 peptide occurred at His34.
(TIF)

## References

[1] Lee JW, Helmann JD. Functional specialization within the Fur family of metalloregulators. Biometals. 2007;20(3–4):485–99. 10.1007/s10534-006-9070-7 . 17216355

[2] Fillat MF. The FUR (ferric uptake regulator) superfamily: diversity and versatility of key transcriptional regulators. Arch Biochem Biophys. 2014;546:41–52. 10.1016/j.abb.2014.01.029 . 24513162

[3] Shin JH, Jung HJ, An YJ, Cho YB, Cha SS, Roe JH. Graded expression of zinc-responsive genes through two regulatory zinc-binding sites in Zur. Proceedings of the National Academy of Sciences of the United States of America. 2011;108(12):5045–50. Epub 2011/03/09. 10.1073/pnas.1017744108 ; PubMed Central PMCID: PMCPmc3064357. 21383173 PMC3064357

[4] Sheikh MA, Taylor GL. Crystal structure of the Vibrio cholerae ferric uptake regulator (Fur) reveals insights into metal co-ordination. Molecular microbiology. 2009;72(5):1208–20. Epub 2009/04/30. 10.1111/j.1365-2958.2009.06718.x . 19400801

[5] Lucarelli D, Russo S, Garman E, Milano A, Meyer-Klaucke W, Pohl E. Crystal structure and function of the zinc uptake regulator FurB from Mycobacterium tuberculosis. J Biol Chem. 2007;282(13):9914–22. Epub 2007/01/11. 10.1074/jbc.M609974200 . 17213192

[6] Dian C, Vitale S, Leonard GA, Bahlawane C, Fauquant C, Leduc D, et al The structure of the Helicobacter pylori ferric uptake regulator Fur reveals three functional metal binding sites. Molecular microbiology. 2011;79(5):1260–75. Epub 2011/01/07. 10.1111/j.1365-2958.2010.07517.x . 21208302

[7] Gilston BA, Wang S, Marcus MD, Canalizo-Hernandez MA, Swindell EP, Xue Y, et al Structural and mechanistic basis of zinc regulation across the E. coli Zur regulon. PLoS biology. 2014;12(11):e1001987 Epub 2014/11/05. 10.1371/journal.pbio.1001987 ; PubMed Central PMCID: PMCPmc4219657. 25369000 PMC4219657

[8] Deng Z, Wang Q, Liu Z, Zhang M, Machado AC, Chiu TP, et al Mechanistic insights into metal ion activation and operator recognition by the ferric uptake regulator. Nature communications. 2015;6:7642 Epub 2015/07/03. 10.1038/ncomms8642 ; PubMed Central PMCID: PMCPmc4506495. 26134419 PMC4506495

[9] Jacquamet L, Traore DA, Ferrer JL, Proux O, Testemale D, Hazemann JL, et al Structural characterization of the active form of PerR: insights into the metal-induced activation of PerR and Fur proteins for DNA binding. Molecular microbiology. 2009;73(1):20–31. Epub 2009/06/11. 10.1111/j.1365-2958.2009.06753.x . 19508285

[10] Dubbs JM, Mongkolsuk S. Peroxide-sensing transcriptional regulators in bacteria. Journal of bacteriology. 2012;194(20):5495–503. Epub 2012/07/17. 10.1128/jb.00304-12 ; PubMed Central PMCID: PMCPmc3458676. 22797754 PMC3458676

[11] Lee JW, Helmann JD. The PerR transcription factor senses H2O2 by metal-catalysed histidine oxidation. Nature. 2006;440(7082):363–7. 10.1038/nature04537 . 16541078

[12] Parent A, Caux-Thang C, Signor L, Clemancey M, Sethu R, Blondin G, et al Single glutamate to aspartate mutation makes ferric uptake regulator (Fur) as sensitive to H2O2 as peroxide resistance regulator (PerR). Angew Chem Int Ed Engl. 2013;52(39):10339–43. 10.1002/anie.201304021 . 23940006

[13] Rey MW, Ramaiya P, Nelson BA, Brody-Karpin SD, Zaretsky EJ, Tang M, et al Complete genome sequence of the industrial bacterium Bacillus licheniformis and comparisons with closely related Bacillus species. Genome biology. 2004;5(10):R77 Epub 2004/10/06. 10.1186/gb-2004-5-10-r77 ; PubMed Central PMCID: PMCPmc545597. 15461803 PMC545597

[14] Voigt B, Schroeter R, Schweder T, Jurgen B, Albrecht D, van Dijl JM, et al A proteomic view of cell physiology of the industrial workhorse Bacillus licheniformis. J Biotechnol. 2014;191:139–49. 10.1016/j.jbiotec.2014.06.004 . 25011098

[15] Lee JW, Helmann JD. Biochemical characterization of the structural Zn2+ site in the Bacillus subtilis peroxide sensor PerR. J Biol Chem. 2006;281(33):23567–78. 10.1074/jbc.M603968200 . 16766519

[16] Heo YJ, Chung IY, Cho WJ, Lee BY, Kim JH, Choi KH, et al The major catalase gene (katA) of Pseudomonas aeruginosa PA14 is under both positive and negative control of the global transactivator OxyR in response to hydrogen peroxide. Journal of bacteriology. 2010;192(2):381–90. 10.1128/JB.00980-09 19933365 PMC2805318

[17] Ji CJ, Kim JH, Won YB, Lee YE, Choi TW, Ju SY, et al Staphylococcus aureus PerR Is a Hypersensitive Hydrogen Peroxide Sensor using Iron-mediated Histidine Oxidation. J Biol Chem. 2015;290(33):20374–86. 10.1074/jbc.M115.664961 26134568 PMC4536443

[18] Won YB, Ji CJ, Cho JH, Lee JW. Mutational Analysis of the Metal-binding Sites of Peroxide Sensor PerR. B Korean Chem Soc. 2010;31(6):1573–6. 10.5012/bkcs.2010.31.6.1573 .

[19] Faulkner MJ, Ma Z, Fuangthong M, Helmann JD. Derepression of the Bacillus subtilis PerR peroxide stress response leads to iron deficiency. Journal of bacteriology. 2012;194(5):1226–35. 10.1128/JB.06566-11 22194458 PMC3294777

[20] Horsburgh MJ, Ingham E, Foster SJ. In Staphylococcus aureus, fur is an interactive regulator with PerR, contributes to virulence, and Is necessary for oxidative stress resistance through positive regulation of catalase and iron homeostasis. Journal of bacteriology. 2001;183(2):468–75. Epub 2001/01/03. 10.1128/jb.183.2.468-475.2001 ; PubMed Central PMCID: PMCPmc94901. 11133939 PMC94901

[21] Rea RB, Gahan CG, Hill C. Disruption of putative regulatory loci in Listeria monocytogenes demonstrates a significant role for Fur and PerR in virulence. Infection and immunity. 2004;72(2):717–27. Epub 2004/01/27. ; PubMed Central PMCID: PMCPmc321596. 14742513 10.1128/IAI.72.2.717-727.2004 PMC321596

[22] Bsat N, Herbig A, Casillas-Martinez L, Setlow P, Helmann JD. Bacillus subtilis contains multiple Fur homologues: identification of the iron uptake (Fur) and peroxide regulon (PerR) repressors. Molecular microbiology. 1998;29(1):189–98. . 9701813 10.1046/j.1365-2958.1998.00921.x

[23] Hantke K. Bacterial zinc uptake and regulators. Current opinion in microbiology. 2005;8(2):196–202. Epub 2005/04/02. 10.1016/j.mib.2005.02.001 . 15802252

[24] Peroza EA, dos Santos Cabral A, Wan X, Freisinger E. Metal ion release from metallothioneins: proteolysis as an alternative to oxidation. Metallomics. 2013;5(9):1204–14. 10.1039/c3mt00079f . 23835914

[25] Traore DA, El Ghazouani A, Jacquamet L, Borel F, Ferrer JL, Lascoux D, et al Structural and functional characterization of 2-oxo-histidine in oxidized PerR protein. Nature chemical biology. 2009;5(1):53–9. Epub 2008/12/17. 10.1038/nchembio.133 . 19079268

[26] Ma Z, Gabriel SE, Helmann JD. Sequential binding and sensing of Zn(II) by Bacillus subtilis Zur. Nucleic Acids Res. 2011;39(21):9130–8. 10.1093/nar/gkr625 21821657 PMC3241647

[27] Ma Z, Faulkner MJ, Helmann JD. Origins of specificity and cross-talk in metal ion sensing by Bacillus subtilis Fur. Molecular microbiology. 2012;86(5):1144–55. 10.1111/mmi.12049 23057863 PMC3508374

[28] Helmann JD. Specificity of metal sensing: iron and manganese homeostasis in Bacillus subtilis. J Biol Chem. 2014;289(41):28112–20. 10.1074/jbc.R114.587071 25160631 PMC4192466

[29] Ma Z, Lee JW, Helmann JD. Identification of altered function alleles that affect Bacillus subtilis PerR metal ion selectivity. Nucleic Acids Res. 2011;39(12):5036–44. 10.1093/nar/gkr095 21398634 PMC3130269

[30] O'Brian MR. Perception and Homeostatic Control of Iron in the Rhizobia and Related Bacteria. Annu Rev Microbiol. 2015;69:229–45. 10.1146/annurev-micro-091014-104432 . 26195304

[31] Ahn BE, Cha J, Lee EJ, Han AR, Thompson CJ, Roe JH. Nur, a nickel-responsive regulator of the Fur family, regulates superoxide dismutases and nickel transport in Streptomyces coelicolor. Molecular microbiology. 2006;59(6):1848–58. 10.1111/j.1365-2958.2006.05065.x . 16553888

[32] Hahn JS, Oh SY, Roe JH. Regulation of the furA and catC operon, encoding a ferric uptake regulator homologue and catalase-peroxidase, respectively, in Streptomyces coelicolor A3(2). Journal of bacteriology. 2000;182(13):3767–74. 10850993 10.1128/jb.182.13.3767-3774.2000 PMC94549

[33] Hahn JS, Oh SY, Chater KF, Cho YH, Roe JH. H2O2-sensitive fur-like repressor CatR regulating the major catalase gene in Streptomyces coelicolor. J Biol Chem. 2000;275(49):38254–60. 10.1074/jbc.M006079200 . 10991944

[34] Schroeter R, Voigt B, Jurgen B, Methling K, Pother DC, Schafer H, et al The peroxide stress response of Bacillus licheniformis. Proteomics. 2011;11(14):2851–66. 10.1002/pmic.201000461 . 21674797

