# Supplementary figures and images for: Bacillus licheniformis Contains Two More PerR-Like Proteins in Addition to PerR, Fur, and Zur Orthologues
Source: PLoS One. 2016 May 13;11(5):e0155539. doi: 10.1371/journal.pone.0155539 (PMC4866751; doi:10.1371/journal.pone.0155539)

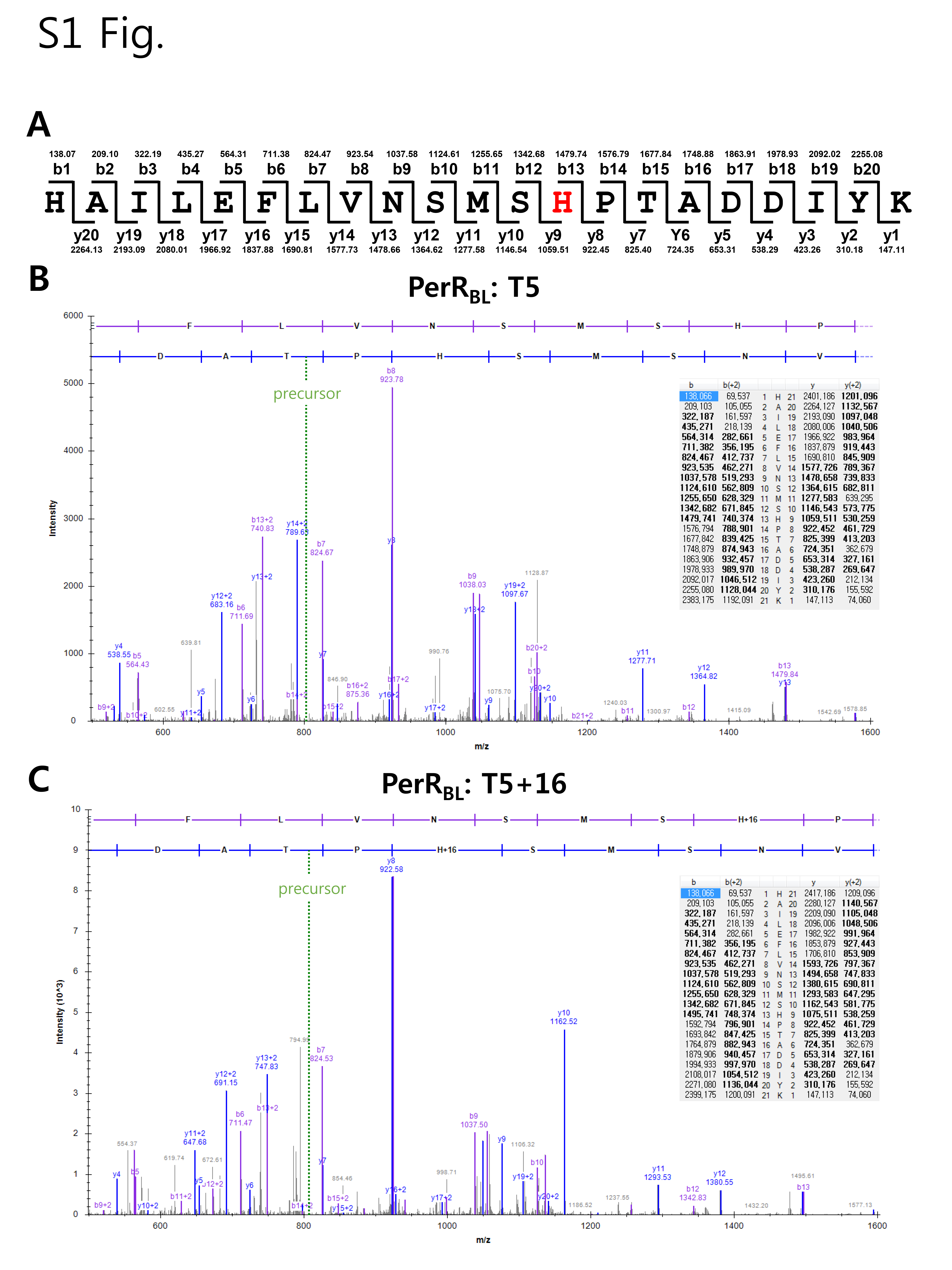

Supplement: S1 Fig — (A) Predicted m/z values of b- and y-ions of unmodified T5 peptide of PerRBL. His37 is shown in red. (B) Tandem MS spectrum of T5 peptide. Triple charged precursor ion ([T5+3H]3+ = 801.85, shown in green) was analyzed by tandem MS. The b- and y-ions are shown in purple and blue, respectively. (C) Tandem MS spectrum of T5+16 peptide. Triple charged precursor ion ([T5+16+3H]3+ = 806.82, shown in green) was analyzed by tandem MS. The b- and y-ions are shown in purple and blue, respectively. The y-ions not containing His37 (y4-y8) appear at the predicted m/z values, whereas the subsequent y-ions containing His37 (y9-y20) have a +16 Da mass shift. Note that almost all the y9- and y10-ions (containing His37 but not Met35) have a +16 Da mass shift. The b-ions not containing His37 (b5-b12) appear at the predicted m/z values, whereas the subsequent b-ions containing His37 (b13-b20) have a +16 Da mass shift. Note that almost all the b10- and b11-ions (containing Met35 but not His37) appear at the predicted m/z values. Taken together, these data indicate that most of the oxidation in T5+16 peptide occurred at His37 rather than Met35. (TIF) [file pone.0155539.s001.tif]

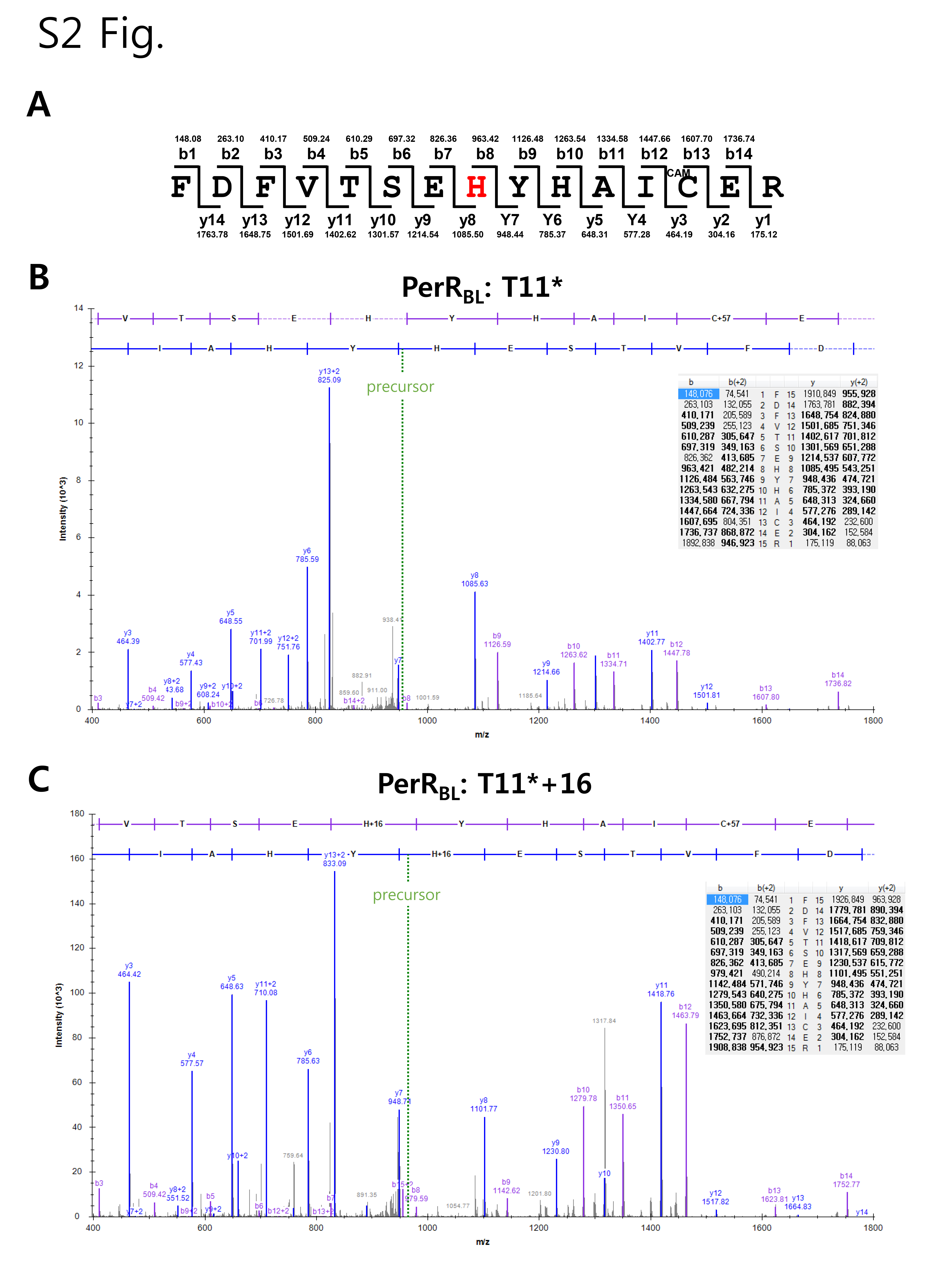

Supplement: S2 Fig — (A) Predicted m/z values of b- and y-ions of T11* peptide (containing carboxyamidomethylated Cys96 residue) of PerRBL. His91 is shown in red. (B) Tandem MS spectrum of T11* peptide. Double charged precursor ion ([T11*+2H]2+ = 956.06, shown in green) was analyzed by tandem MS. The b- and y-ions are shown in purple and blue, respectively. (C) Tandem MS spectrum of T11*+16 peptide. Double charged precursor ion ([T11*+16+2H]2+ = 964.42, shown in green) was analyzed by tandem MS. The b- and y-ions are shown in purple and blue, respectively. The y-ions not containing His91 (y3-y7) appear at the predicted m/z values, whereas the subsequent y-ions containing His91 (y8-y14) have a +16 Da mass shift. The b-ions not containing His91 (b3-b7) appear at the predicted m/z values, whereas the subsequent b-ions containing His91 (b8-b14) have a +16 Da mass shift. These data indicate that the oxidation in T11*+16 peptide occurred at His91. (TIF) [file pone.0155539.s002.tif]

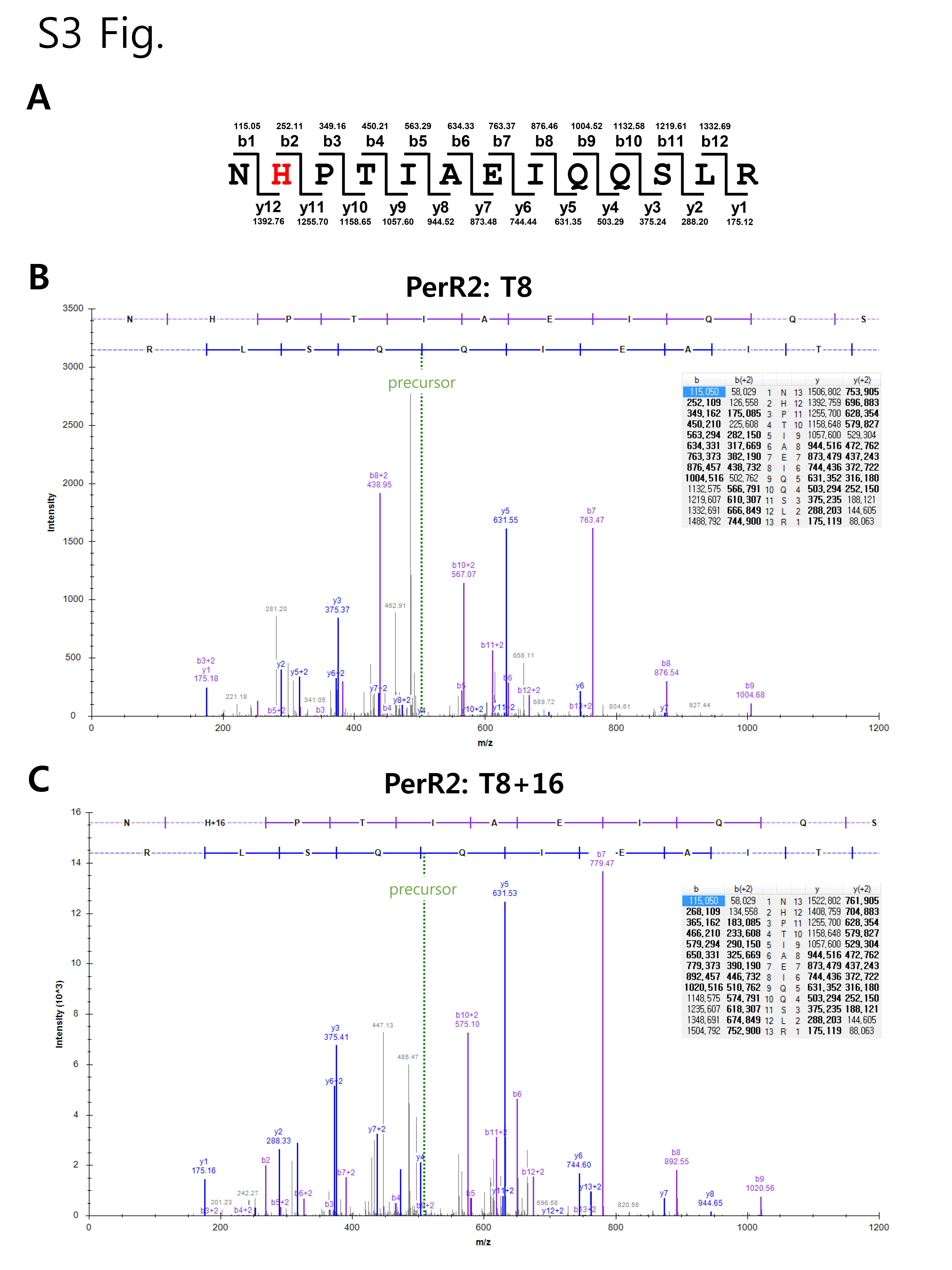

Supplement: S3 Fig — (A) Predicted m/z values of b- and y-ions of T8 peptide of PerR2. His39 is shown in red. (B) Tandem MS spectrum of T8 peptide. Triple charged precursor ion ([T8+3H]3+ = 503.20, shown in green) was analyzed by tandem MS. The b- and y-ions are shown in purple and blue, respectively. (C) Tandem MS spectrum of T8+16 peptide. Triple charged precursor ion ([T8+16+3H]3+ = 509.16, shown in green) was analyzed by tandem MS. The b- and y-ions are shown in purple and blue, respectively. The y-ions not containing His39 (y1-y11) appear at the predicted m/z values, whereas the subsequent y-ions containing His39 (y12-y13) have a +16 Da mass shift. The b-ions containing His39 (b2-b13) have a +16 Da mass shift. These data indicate that the oxidation in T8+16 peptide occurred at His39. (TIF) [file pone.0155539.s003.tif]

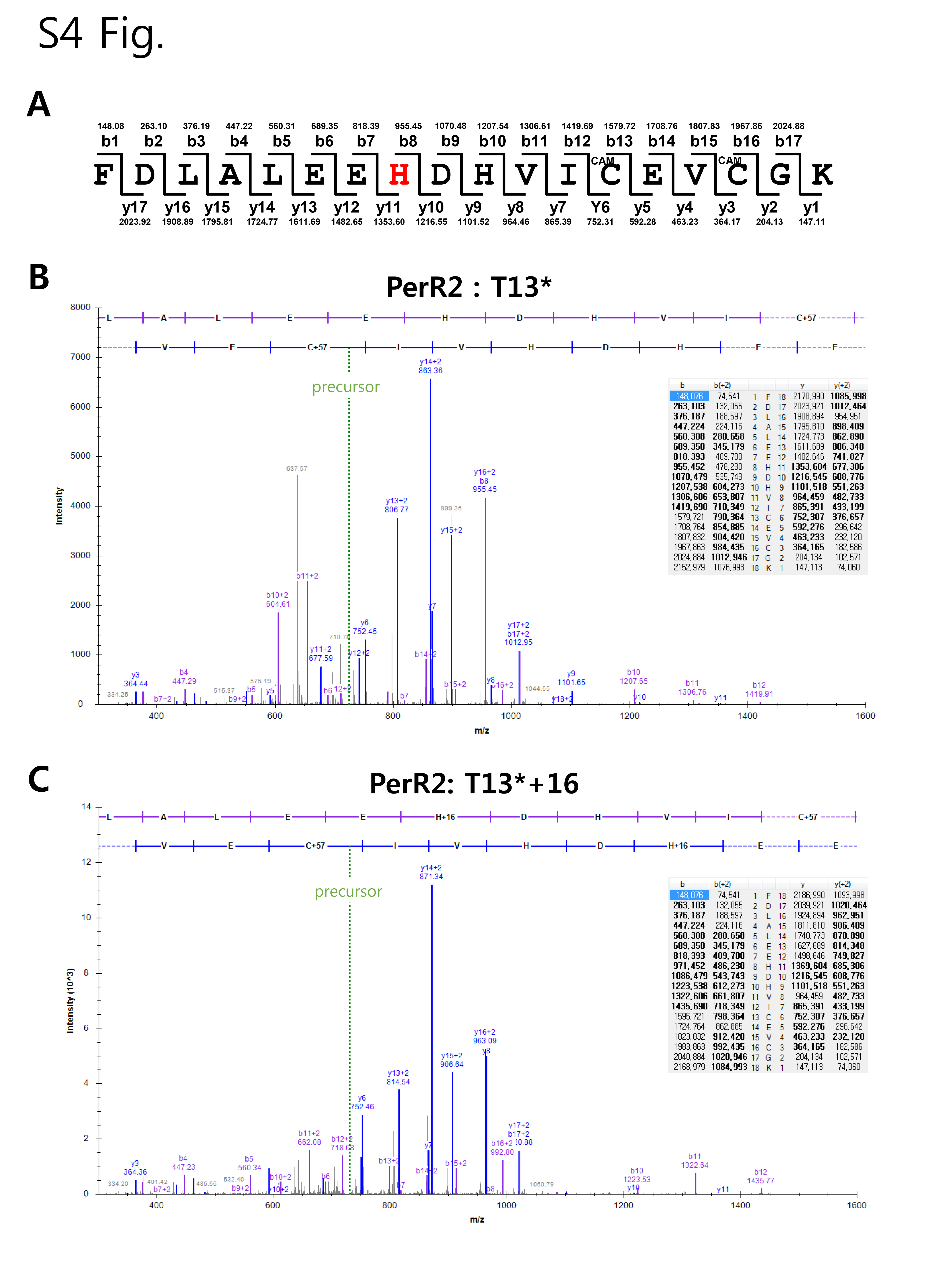

Supplement: S4 Fig — (A) Predicted m/z values of b- and y-ions of T13* peptide (containing carboxyamidomethylated Cys97 and Cys100 residues) of PerR2. His92 is shown in red. (B) Tandem MS spectrum of T13* peptide. Triple charged precursor ion ([T13*+3H]3+ = 724.93, shown in green) was analyzed by tandem MS. The b- and y-ions are shown in purple and blue, respectively. (C) Tandem MS spectrum of T13*+16 peptide. Triple charged precursor ion ([T13*+16+3H]3+ = 730.05, shown in green) was analyzed by tandem MS. The b- and y-ions are shown in purple and blue, respectively. The y-ions not containing His92 (y3-y10) appear at the predicted m/z values, whereas the subsequent y-ions containing His92 (y11-y17) has a +16 Da mass shift. The b-ions not containing His92 (b3-b7) appear at the predicted m/z values, whereas the subsequent b-ions containing His92 (b8-b16) has a +16 Da mass shift. These data indicate that the oxidation in T8+16 peptide occurred at His92. (TIF) [file pone.0155539.s004.tif]

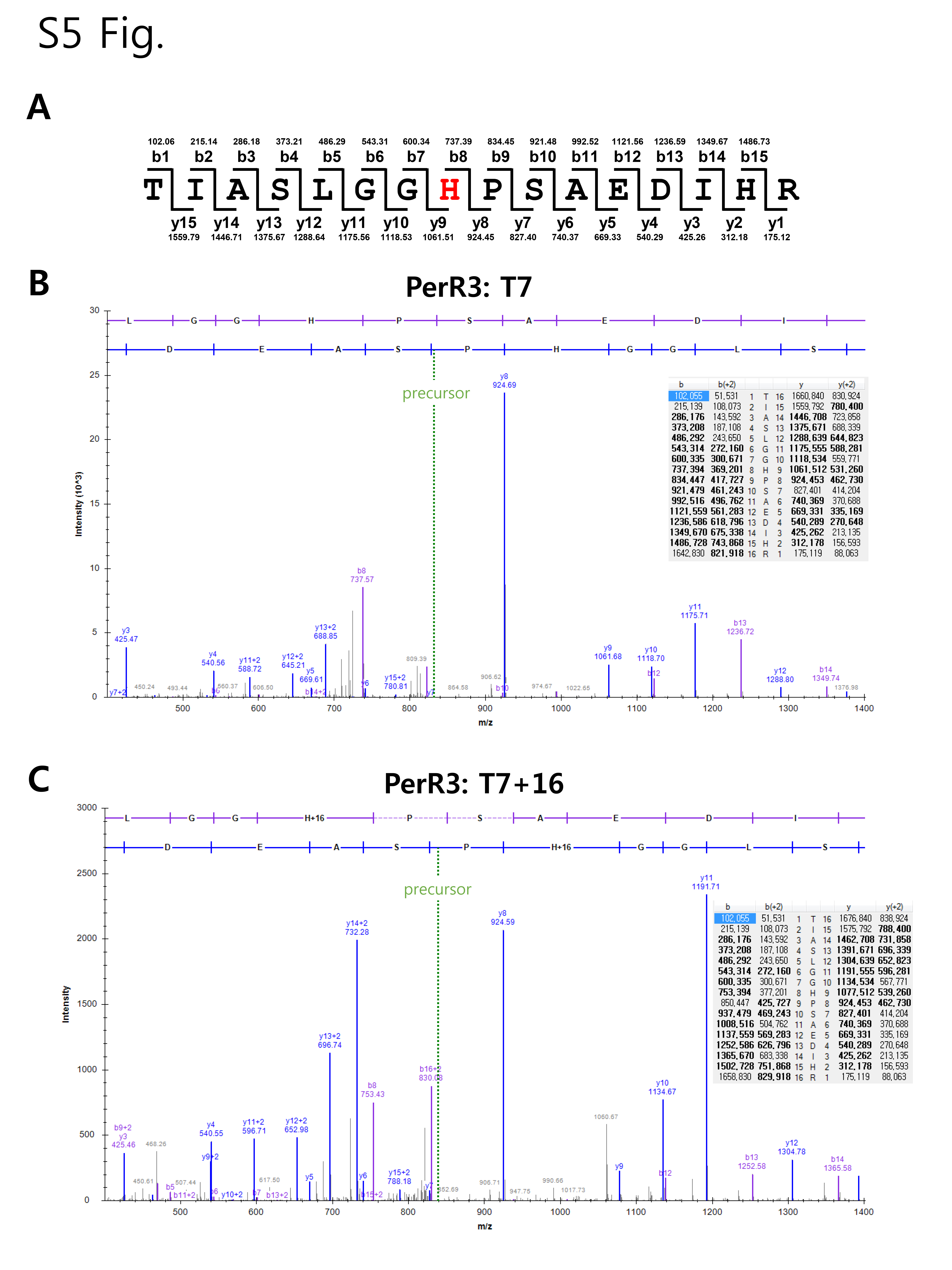

Supplement: S5 Fig — (A) Predicted m/z values of b- and y-ions of T7 peptide of PerR3. His34 is shown in red. (B) Tandem MS spectrum of T7 peptide. Double charged precursor ion ([T7+2H]2+ = 831.40, shown in green) was analyzed by tandem MS. The b- and y-ions are shown in purple and blue, respectively. (C) Tandem MS spectrum of T7+16 peptide. Double charged precursor ion ([T7+16+2H]2+ = 839.29, shown in green) was analyzed by tandem MS. The b- and y-ions are shown in purple and blue, respectively. The y-ions not containing His34 (y3-y8) appear at the predicted m/z values, whereas the subsequent y-ions containing His34 (y9-y15) has a +16 Da mass shift. The b-ions not containing His34 (b5-b7) appear at the predicted m/z values, whereas the subsequent b-ions containing His34 (b8-b16) has a +16 Da mass shift. These data indicate that the oxidation in T7+16 peptide occurred at His34. (TIF) [file pone.0155539.s005.tif]
